# Effect of oral isotretinoin on muscle strength in patients with acne vulgaris: a prospective controlled study

**DOI:** 10.1186/s40360-021-00483-0

**Published:** 2021-03-20

**Authors:** Cevriye Mülkoğlu, Nermin Karaosmanoğlu

**Affiliations:** 1 Department of Physical Medicine and Rehabilitation, Health Sciences University Ankara Training and Research Hospital, Ulucanlar Street, Ankara, Turkey; 2 Department of Dermatology, Health Sciences University Ankara Training and Research Hospital, Ankara, Turkey

**Keywords:** Isotretinoin, Muscle strength, Isokinetic, Creatinine phosphokinase

## Abstract

**Background:**

Musculoskeletal side effects related to isotretinoin are frequently reported. This study aimed to investigate the effect of oral isotretinoin treatment on muscle strength. Our second aim was to evaluate whether there was a correlation between the serum creatine phosphokinase (CPK) level, a specific marker of muscle breakdown, and muscle strength.

**Methods:**

This study included 30 patients who presented to our hospital and were started on oral isotretinoin treatment for acne vulgaris and 30 patients in the control group who were given local treatment. Age, sex, height and weight of the patients were recorded, and the body mass index (BMI) was calculated. The hamstring and quadriceps muscle strengths of the non-dominant side were evaluated in all patients using an isokinetic dynamometer, and the peak torque (PT) values ​​were recorded. In the isotretinoin group, isokinetic measurements were performed again in those that completed six-month drug treatment and compared with the initial PT values.

**Results:**

The two groups were similar in terms of age, sex, and BMI (*p* > 0.05). There was no significant difference between the isotretinoin and control groups in terms of muscle strength at the beginning of the treatment (*p* > 0.05). No significant change was observed in hamstring and quadriceps PT values in the isotretinoin group after 6 months of treatment compared to baseline (*p* > 0.05). No statistically significant correlation was found between the serum CPK level and hamstring and quadriceps muscle strength (*p* > 0.05).

**Conclusion:**

Oral isotretinoin doesn’t alter muscle strength. There is no relationship between the serum CPK levels and muscle strength.

## Key messages

1. The musculoskeletal side effects of isotretinoin include arthralgia, myalgia, sacroiliitis, diffuse idiopathic skeletal hyperostosis, ligament and tendon calcification
2. It was shown that 15 to 50% of the patients with isotretinoin-induced myalgia, had elevated serum level of creatinine phosphokinase (CPK)
3. The effects of isotretinoin on muscle strength are not yet fully understood
4. We found that systemic isotretinoin did not cause any dysfunction in hamstring and quadriceps muscle strength on 6 months of treatment in patients with acne vulgaris
5. There was no relationship between serum CPK levels and muscle strength

## Background

Isotretinoin is a vitamin A derivative frequently used in the resolution of treatment-resistant, moderate to severe acne vulgaris [1, 2]. Some of the musculoskeletal side effects of isotretinoin are arthralgia, myalgia, sacroiliitis, diffuse idiopathic skeletal hyperostosis, and ligament and tendon calcification [3–5]. In 15 to 50% of patients with isotretinoin-induced myalgia, an elevated serum level of creatinine phosphokinase (CPK) has been shown [6]. However, the effects of isotretinoin on muscle strength are not yet fully understood. To our knowledge, the literature contains only one study investigating this subject using an isokinetic dynamometer. In that study, the hamstring and quadriceps muscle strength of 26 patients that received isotretinoin for acne vulgaris and 26 control patients who did not receive systemic medication were evaluated at the beginning and at 3 months into treatment [3]. The advantages of our study were the follow-up period being increased from 3 to 6 months and the additional investigation of the relationship between the blood CPK levels and muscle strength.

The primary aim of our study was to evaluate and compare the knee muscle strengths of 30 patients that were planned to use isotretinoin for the treatment of acne vulgaris and 30 patients who were given local treatment, based on isokinetic dynamometer measurements undertaken at the beginning and 6 months after treatment. Our second aim was to investigate whether there was a correlation between the isokinetic parameters and the serum CPK levels.

## Methods

### Study design and setting

Patients with acne vulgaris that presented to Ankara Training and Research Hospital were included in this prospective controlled study. The study was conducted between September 2018 and October 2019. All participants were informed about the study, and their informed consent was obtained. The study protocol was approved by the ethics committee of our hospital. Our study adheres to CONSORT guidelines.

### Participants

The patients who applied to the outpatient clinic with acne vulgaris were examined by the same dermatologist. The dermatologist assessed the skin lesions and determined which treatment (systemic isotretinoin or local treatment) will be initiated, depending on the severity of acne vulgaris. Patients were allocated to either isotretinoin group or control group. Thirty patients with newly diagnosed acne vulgaris and scheduled for oral isotretinoin treatment were included in the isotretinoin group, and 30 who will be given local treatment were allocated to control group. Before starting these treatments, the dermatologist sent the patients to a physical medicine and rehabilitation specialist for isokinetic testing. The physical medicine and rehabilitation specialist who performing the isokinetic evaluation was blind which treatment will be initiated to the patients. The age, sex, height and weight of the patients were recorded. Body mass index (BMI) was calculated. Isotretinoin treatment was started at a dose of 1–2 mg/kg/day with the aim of reaching a cumulative dose of 120–150 mg/kg over 4 to 6 months. It was questioned that whether the subjects made regular exercises or sport activities in their daily life, when taking their history. We made some adjustments for patients’ life style and habits. The patients who will be initiated isotretinoin were informed about avoiding heavy activities and intense sports during the treatment.

### Inclusion criteria

Inclusion criteria were being aged 18–45 years and receiving isotretinoin treatment for the isotretinoin group and not having used isotretinoin within the last year for the control group. The exclusion criteria were chronic kidney or liver disease, uncontrolled hypertension, heart failure, malignancy, thyroid and bone diseases (e.g., hyperparathyroidism and osteomalacia), use of drugs that may affect skeletal metabolism (e.g., corticosteroids, heparin, and anticonvulsants), and a history of trauma and/or surgery in the lower extremities. Patients with other skin conditions and muscular disorders, using bone and muscular supplements, overweight and obese patients, and athletes or subjects who do frequent training were also excluded. Patients who discontinued or terminated their isotretinoin treatment were not included in the study.

### Tests and measurements

Serum CPK analysis was requested at the initial visit and during the monthly follow-up visits from the patients in the isotretinoin group. The normal CPK value for adults was accepted as lower 170 IU/L.

The hamstring and quadriceps muscle strength of the patients was evaluated by the same physical therapy and rehabilitation specialist by using an isokinetic dynamometer (Biodex System 4 Pro; Biodex Corporation, Shirley, NY, USA). Isokinetic measurements were applied to the patients in the treatment group twice: at the beginning of the treatment and after 6 months of medication use while the patients in the control group were administered local treatment only at the beginning. The lower extremity concentric muscles (hamstring and quadriceps) on the non-dominant side were evaluated in all participants at angular velocities of 60° and 120°/sec. This evaluation consisted of five times of submaximal warm-up period followed by the isokinetic test protocol, 10 repetitions at 60°/sec angular velocity at 20-s intervals of rest, followed by 15 repetitions at an angular velocity of 120°/sec. The average concentric hamstring and quadriceps peak torque (PT) values ​​were recorded as Newton meters (Nm). Isokinetic dynamometer and the PT values reflect muscle damaging effect of isotretinoin at clinical level.

### Statistical analysis

Data analysis was conducted using SPSS version 21.0. The Shapiro-Wilk test was used to evaluate the normal distribution of the data. The normally distributed data were expressed as mean ± standard deviation (SD), and those without normal distribution were expressed as median. The independent samples t-test was carried out to compare the baseline parameters between the isotretinoin and control groups, and the Mann-Whitney-U test was utilized to compare non-parametric data. The paired samples t-test and Wilcoxon signed-rank test were used to compare the isokinetic parameters at the baseline and 6 months after treatment. The relationship between the non-parametric CPK level and hamstring and quadriceps PT values was evaluated using the Spearman correlation analysis. A *p* value of < 0.05 was considered statistically significant.

## Results

### Study population

**Fig. 1:**
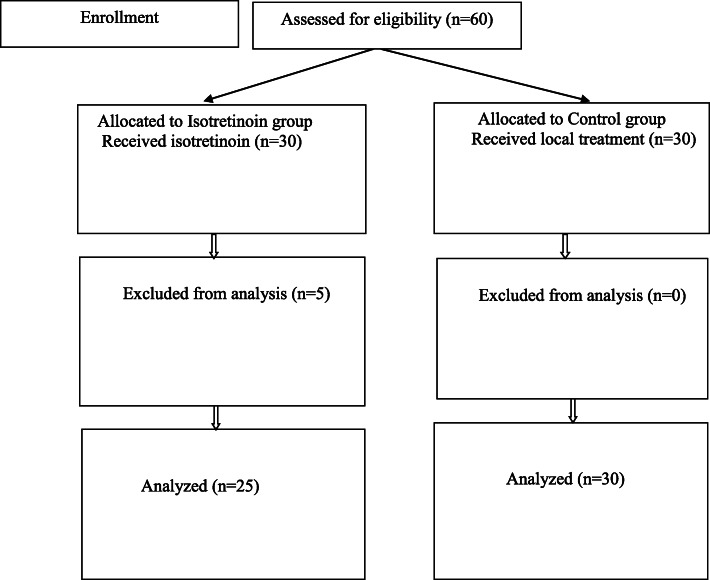
Flowchart of the subjects

Five of the 30 patients that were started on systemic isotretinoin were excluded from the study due to side effects (sacroiliitis in one, low back pain in two, and arthralgia in two), and the study was completed with 25 (19 females and 6 males) patients in this group. The flowchart of the patients recruitment process is given in Fig. 1. In the isotretinoin group, the median age was 19 (18–27) years, the mean BMI was 21.5 ± 0.56 kg/cm^2^, and the mean cumulative drug dose was 5660 ± 913.78 mg. In the control group (23 females and 7 males), the median age of the patients was 19 (18–28) years, and the mean BMI was 22.6 ± 2.5 kg/cm^2^. No statistically significant difference was found between the two groups in terms of age, sex, and BMI (*p* > 0.05). The demographic characteristics of the study population are presented in Table 1.

**Table 1:** Demographic characteristics and baseline isokinetic assessment results of the study groups

	Isotretinoin group (*n* = 25)	Control group (*n* = 30)	*p* value
Sex			
Female (n) (%)	19 (74)	23 (76.6)	0.93
Male (n) (%)	6 (24)	7 (23.4)	
Age (median) (min-max)	19 (18–27)	19 (18–28)	0.90
BMI (mean ± SD)	21.5 ± 2.8	22.6 ± 2.5	0.15
CPK IU/l (mean ± SD)	82.6 ± 39.1	84.7 ± 30.8	0.82
Mean concentric quadriceps PT			
(Nm) (SD)			
60°/s AV	111.2 ± 33.5	105.1 ± 39.8	0.55
120°s AV	77.0 ± 26.1	76.7 ± 29.0	0.97
Mean concentric hamstring PT			
(Nm) (SD)			
60°/s AV	66.1 ± 15.8	62.3 ± 15.7	0.39
120°/s AV	54.0 ± 12.2	53.2 ± 15.0	0.81

*BMI* Body mass index, *SD* Standard deviation, *IQR* Interquartile range, *CPK* Creatinine phosphokinase, *PT* Peak torque, *Nm* Newton-meter, *AV* Angular velocity

### Isokinetic evaluation at baseline

According to the initial isokinetic measurement results, there was no significant difference between the isotretinoin and control groups in relation to the concentric hamstring and quadriceps PT values (*p* > 0.05). The baseline isokinetic testing results of the patients in both groups are given in Table 1.

### Isokinetic evaluation at sixth-month

When compared with the initial values, no statistically significant difference was observed between the hamstring and quadriceps PT values obtained at the sixth month of isotretinoin treatment (*p* > 0.05). Table 2 presents the results of the isokinetic evaluation of the patients in the isotretinoin group at the beginning and 6 months into treatment.

**Table 2:** Baseline and sixth-month isokinetic assessment results of the isotretinoin group

	Baseline	Sixth-month	*p* value
Mean concentric quadriceps PT (Nm) (SD)			
60°/s AV	111.2 ± 33.5	116.8 ± 33.1	0.55
120°/s AV	77.0 ± 26.1	81.9 ± 23.1	0.48
Mean concentric hamstring PT (Nm) (SD)			
60°/s AV	66.1 ± 15.8	67.9 ± 17.0	0.70
120°/s AV	54.0 ± 12.2	54.1 ± 15.1	0.99

*PT* Peak torque, *AV* Angular velocity, *SD* Standard deviation, *Nm* Newton-meter. *p* < 0.05 shows statistical significance

### Serum CPK values

The mean serum CPK value was 82.6 ± 39.1 IU/l in the isotretinoin group, and 84.7 ± 30.8 IU/l in the control group. There was no significant difference between the groups in terms of serum CPK levels (*p* = 0.82). Elevated serum CPK levels were observed in only 4 of the 25 patients in the isotretinoin group during the follow-up visits, one of them had myalgia, the others were asymptomatic.

### Correlation between serum CPK levels and muscle strength

We evaluated the relationship between the serum CPK levels and muscle strength. No statistically significant correlation was found between the quadriceps and hamstring PT values ​​and the serum CPK levels (*p* > 0.05). The results of the correlation analysis between the serum CPK levels and isokinetic parameters are shown in Table 3.

**Table 3:** The results of correlation analysis between serum CPK and isokinetic parameters

	Serum CPK level (IU/l)
**Concentric quadriceps PT (Nm)**	
60°/s AV	r = 0.150 *p* = 0.283
120°/s AV	r = 0.227 *p* = 0.102
**Concentric hamstring PT (Nm)**	
60°/s AV	r = 0.192 *p* = 0.167
120°/s AV	r = 0.195 *p* = 0.162

*CPK* Creatinine phosphokinase, *PT* Peak torque, *Nm* Newton-meter, *AV* Angular velocity, *r* rho correlation coefficient. Statistically significant at *p* < 0.05

## Discussion

Myalgia, muscle tenderness, and stiffness are among the musculoskeletal side effects frequently reported during systemic isotretinoin treatment [6, 7]. These symptoms are usually mild and quickly reversible with the discontinuation of isotretinoin [8, 9]. Acute myopathy or rhabdomyolysis is rarely observed as a complication during oral isotretinoin treatment [10–12]. The side effects of isotretinoin on muscles have been reported in the literature were mostly as case reports [10–12]. Hodak et al. described 2 young men (aged 16 and 20) with nodulocystic acne who have developed clinical and electromyographic changes of muscle damage during isotretinoin treatment. In one patient the muscle damage was confirmed by histological and ultrastructural findings. They showed that isotretinoin may induce reversible skeletal muscle damage [10].

Elevated CPK blood levels and muscular symptoms and have been reported in 15–50% of patients receiving isotretinoin for acne [6, 12, 13]. Trauner et al. reported a male patient who aged 49 and was developed a significant elevation in blood CPK level after beginning treatment with isotretinoin for dissecting cellulitis on the scalp. The patient was asymptomatic and had no history of recent musculoskeletal injuries, myalgias, surgical procedures, excessive exercise, or intramuscular injections. The authors mentioned that isotretinoin was discontinued within 24 h of the 5-week routine follow-up laboratory evaluation and serum CPK levels quickly declined after stopping isotretinoin and normalized within 15 days [12].

Although there are various case reports on oral isotretinoin and muscle side effects [10–12]; there seems to be an inadequacy in terms of original research. The effect of isotretinon on muscle strength was evaluated in only one study. In that study, Yıldızgören et al. evaluated 26 patients that received isotretinoin for acne vulgaris and 26 control patients who did not receive systemic medication in terms of the hamstring and quadriceps muscle strength of at the beginning and at 3 months into treatment. They reported no significant change in hamstring and quadriceps muscle strength when they compared the baseline and third-month values in the isotretinoin group [3]. The superiority of our study is that we evaluated muscle strength at 6 months of isotretinoin treatment and also investigated the correlation between the serum CPK levels and muscle strength of hamstring and quadriceps. When we compared the isokinetic parameters at the beginning of isotretinoin treatment and at the sixth treatment month, we found that oral isotretinoin did not have a significant effect on isokinetic hamstring and quadriceps muscle strength. In addition, no significant correlation was observed between the serum CPK level and muscle strength. In light of these results, we can state that oral isotretinoin does not cause any dysfunction in the lower extremity muscle strength after 6 months of use in patients with acne vulgaris. Furthermore, the serum CPK level was not correlated with lower extremity muscle strength. These non-significant results in this study may be related to the small numbers of the patients in the groups. There is clear need for further studies with a larger patient groups.

It is known that CPK, which is a specific marker of muscle breakdown, increases in patients receiving oral isotretinoin treatment, especially in those who undertake intense physical exercises (with or without muscle symptoms and signs) [10, 14–16]. A high CPK level shows severe muscle damage and is generally associated with the discharge of myoglobin from muscles [11]. In patients treated with oral isotretinoin, concurrent intense physical activity or viral infections may cause increased serum CPK levels (without myopathy) due to cytokine-mediated muscle cell damage [6, 14–17]. Kaymak et al. evaluated 89 patients who were treated with isotretinoin for moderate or severe acne. They investigated serum CPK levels at the initial visit and during the monthly visits and observed high CPK levels in only five patients during treatment period. Only one of these had myalgia and four had no symptom [13]. In current study, at the beginning of the study the two groups were similar in terms of serum CPK levels. Increased CPK levels were observed in only 4 of the 25 patients, in the isotretinoin group. One of 4 patients had myalgia and the others were asymptomatic.

Landau et al. reported that CPK levels more than 5000 IU/l in six out of seven patients who have physical activity or intramuscular injection, before the blood testing. It has been claimed that exercise in patients using isotretinoin may cause elevated serum CPK levels [15]. Tillman et al. reported that the patient’s recent exercise level was the major factor on CPK value [17]. Bettoli et al. examined CPK levels in 63 patients receiving isotretinoin for nodulocystic acne. There was increased CPK levels in 10 of 63 patients (16%). It was found that the most patients (8/10) have elevations less than three times the normal values and only one patient had myalgia [18]. In present study, 4 patients had mildly elevated CPK levels in isotretinoin group. One of them had myalgia, the others had no musculoskeletal symptoms. Also, serum CPK levels were not related with muscle strength in patients using isotretinoin. Even in patients with high serum CPK levels, muscle strength may be normal. Elevated serum CPK levels with or without muscular symptoms in patients receiving isotretinoin seems to be a benign condition. The results of this study show that the periodic blood testing for CPK levels is unnecessary in all patients on isotretinoin treatment. The serum CPK levels may be requested from the patients only with severe muscle pain.

## Conclusion

Oral isotretinoin did not cause any dysfunction in hamstring and quadriceps muscle strength on 6 months of treatment in patients with acne vulgaris. There was no relationship between serum CPK levels and muscle strength.

## Limitations

There are some limitations of this study. The most important limitation is the small number of patients. We had to exclude 5 patients in the isotretinoin group due to side effects of the drug and we completed the study with total of 55 patients. The another limitation is that we evaluated the participants only with isokinetic device for measuring muscle strength, but we did not use other measurement tools. We assessed only the lower extremity muscles, quadriceps and hamstring, but did not evaluate the other muscles. Despite its limitations, to the best of our knowledge, this is the longest-term prospective controlled study from a single-center, investigating the effect of isotretinoin on muscle strength.

## Data Availability

The datasets generated and/or analysed during the current study are not publicly available due to the patients’ privacy but are available from the corresponding author on reasonable request.

## Abbreviations

CPK: Creatinine phosphokinase
BMI: Body mass index
PT: Peak torque
